# Rail Track Detection and Projection-Based 3D Modeling from UAV Point Cloud

**DOI:** 10.3390/s20185220

**Published:** 2020-09-13

**Authors:** Shima Sahebdivani, Hossein Arefi, Mehdi Maboudi

**Affiliations:** 1 School of Surveying and Geospatial Eng., College of Eng., University of Tehran, Tehran 1439957131, Iran; sh.sahebdivani@ut.ac.ir; 2 Institute of Geodesy and Photogrammetry, Technische Universität Braunschweig, 38106 Braunschweig, Germany; m.maboudi@tu-bs.de

**Keywords:** railroad, 3D modeling, UAV-based point cloud, railway detection, projection-based modeling, RANSAC

## Abstract

The expansion of the railway industry has increased the demand for the three-dimensional modeling of railway tracks. Due to the increasing development of UAV technology and its application advantages, in this research, the detection and 3D modeling of rail tracks are investigated using dense point clouds obtained from UAV images. Accordingly, a projection-based approach based on the overall direction of the rail track is proposed in order to generate a 3D model of the railway. In order to extract the railway lines, the height jump of points is evaluated in the neighborhood to select the candidate points of rail tracks. Then, using the RANSAC algorithm, line fitting on these candidate points is performed, and the final points related to the rail are identified. In the next step, the pre-specified rail piece model is fitted to the rail points through a projection-based process, and the orientation parameters of the model are determined. These parameters are later improved by fitting the Fourier curve, and finally a continuous 3D model for all of the rail tracks is created. The geometric distance of the final model from rail points is calculated in order to evaluate the modeling accuracy. Moreover, the performance of the proposed method is compared with another approach. A median distance of about 3 cm between the produced model and corresponding point cloud proves the high quality of the proposed 3D modeling algorithm in this study.

## 1. Introduction

Railways are highly efficient means of intra-city and suburban transportation in developed countries, and millions of people around the world use railways to travel. Given the importance of railways in the economic and social development of societies, it is essential to maintain their safety. In order to maintain rail safety standards, many countries make regular inspections to prevent possible accidents due to the fatigue of rail lines. One of the most important and useful tools for this purpose, which has received much attention in recent years, is the use of photogrammetric products, including modeling the railway environment, especially rail lines. These products can be used as the basis for inspection and monitoring purposes. Three-dimensional modeling of rail tracks can be used accurately and efficiently to monitor the potential hazards of railway infrastructure [1,2]. The advantages of using 3D rail models include identifying distortions and deformation of tracks, collision testing, measurement of clearance by simulating railway cars across the route, rapid response to violations, and the creation of an up-to-date database to effectively maintain the railways [1].

In the current research, we intend to detect and model rail lines using photogrammetric point clouds, which have been less discussed in the literature. Despite the various tools for generating 3D data, stereo matching of overlapped images can be a cost-effective and accurate method of producing dense point clouds that include spectral information for better analysis of rail environments. In order to generate the 3D model of the railway, a projection-based approach is proposed based on the main direction of the rail tracks. The overall direction or orientation of the rail tracks is the basis for the projection of the 3D point clouds onto a plane perpendicular to the rail track direction. After the projection step, i.e., eliminating one dimension (here, y-direction), the 3D point space is reduced to the 2D space. Then, the modeling procedure is continued on 2D space by robust fitting a 2D model to the projected points.

By employing a projection-based algorithm, the 3D point cloud is projected onto the plane perpendicular to the main orientation of the rail segment and, in fact, we eliminate one dimension and therefore we further process the modeling in 2D space instead of 3D space. Besides the benefits of using unmanned aerial vehicle (UAV) data for generating 3D information, using UAV images and generated 3D data, especially for rail detection and tracking, also has some challenges. The most dominant challenges are noise and incompleteness of the generated 3D data. The proposed projection-based approach increases the inlier ratio and alleviates the impact of incompleteness of the data on the model fitting result and increases the robustness of the algorithm against noise on the photogrammetric point cloud. Another innovative strategy in our rail track reconstruction approach is that, by considering the local linearity of the railroad structure, the extrusion of a 2D model to a 3D model is performed in such a way that the whole part of the railroad under investigation is split into some locally linear and smoothly connected patches. Last, but not least, the 3D to 2D projections lead to an increase in the efficiency and processing speed of the proposed approach.

The remainder of the paper is structured as follows: A brief research background of railway detection and modeling is given in Section 2. Next, our methodology is explained in Section 3. Section 4 handles the implementation of the proposed method, followed by evaluation and analyses of our results. A conclusion and discussion about the results are presented in Section 5.

## 2. Background

Advances in technology and the demand for effective and better rail monitoring have increased the use of computer vision methods [3]. As a result, many studies have been performed using these techniques in order to recognize and inspect railway tracks. In most of these studies, the recorded images or videos (using a camera mounted on a train or automatic vehicle) and their analysis are used to help the driver assistance system [4,5,6,7] or automatically detect obstacles along the tracks by extracting railway lines [8]. In recent years, with the advancement of technologies such as GPS, laser scanners (installed on trains, land, or air), mobile mapping systems, and drones, a wide range of rail monitoring systems have been offered to inspectors. A short description of the recent systems is given as follows:

### 2.1. Laser Scanning

A laser scanning system provides accurate three-dimensional measurements of the objects in a railroad environment. The generated point cloud is a common tool for detecting and reconstructing the rail tracks [2]. Bagger et al. [9], for example, used the fusion of an orthoimage and a dense point cloud of an airborne laser scanner for reconstructing the central line of railroad tracks. For this purpose, using object-based image analysis, the rail path mask is identified in the orthoimage. This spatial information is then combined with altitude information to classify laser points. Finally, by extracting the features based on the random sample consensus (RANSAC) algorithm [10], the centerline of the rail track is reconstructed. In 2013, Elberink et al. [11] proposed a method for detecting and modeling rails using mobile laser scanner data. They first detected the rail tracks, based on their properties such as their relative height and position to other objects and their linearity, and then fitted a rail piece model to the points. Next, the position and orientation parameters were estimated using the Markov chain Monte Carlo (MCMC) algorithm. In 2014, Zhu et al. employed a combination of both mobile and airborne laser scanner data to model all objects in a rail environment, such as rails, buildings, trees, and power cables [12]. Jwa and Sohn [1] also proposed a method to reconstruct the three-dimensional model of rail tracks using mobile LIDAR data. In this method, the railway track trajectory is first estimated, and then the points of tracks are detected through the Bayesian decision process. Afterward, the points on the rail head are segmented by the region growing method, and next the initial track model is reconstructed by a third-degree polynomial function. The Kalman filter finally optimizes the initial model parameters. Arastounia and Elberink [13] utilized mobile laser scanner data to classify rail environment objects into three classes of rail track, contact cable, and catenary cable. They first made a coarse classification based on the object’s height and then separated the points corresponding to the track bed. The candidate rail points were then identified by detecting the height jumps on the track bed, and non-rail points were deleted by template matching in the 2D space. Additionally, in 2017, Arastounia classified the point cloud of a rail environment using mobile laser scanning (MLS) and terrestrial laser scanning (TLS) datasets and identified the rail tracks that could be used as the basis for modeling and inspecting rail lines [14].

### 2.2. Photogrammetric Point Cloud

Laser scanner technology is well known as a reliable and accurate method in the production of high-resolution point clouds, but it has limitations in the production of spectral features of objects. Additionally, the equipment is usually costly. In contrast, photogrammetric methods for producing point clouds are very cost-effective and can be created and processed using different types of cameras and software. However, to achieve three-dimensional points, they need computational processes that reduce their automation. It is also problematic in shady and dark areas [15]. Today, the use of unmanned aerial vehicles (UAVs) is one of the techniques accepted by experts to achieve accurate, high-quality, and fast mapping data [16]. In recent years, photogrammetric point clouds and especially UAV point clouds have been used in different modeling and reconstruction applications [17,18,19], and UAV point clouds are used to obtain surveying data for analyzing, detecting and reconstructing railways. By using UAV technology, rail mapping projects can be controlled from outside the railway area, thus preventing the disruption of rail services as well as saving people from danger [20]. Although this technology has been commercially developed in the field of railways, few research studies have been conducted to fully uncover its abilities [21]. Among these research works, one can mention the application of drones in monitoring railway tracks by detecting the vanishing point in the images [22]. Singh et al. [23] applied edge detection algorithms in UAV images to identify rail lines and calculated the geometric parameter of the rail gauge. Additionally, in 2019, Banic et al., by processing the video frames taken by a UAV using the edge detection operator and the K-nearest neighbor (KNN) classifier, identified rail tracks for automatic inspection [24]. To the best of our knowledge, there has been no previous study on the reconstruction of rail tracks using point clouds obtained from photogrammetric methods and the use of UAVs. Therefore, in this study, we investigate the performance of this type of three-dimensional data in rail line modeling.

## 3. Methods

**Figure 1:**
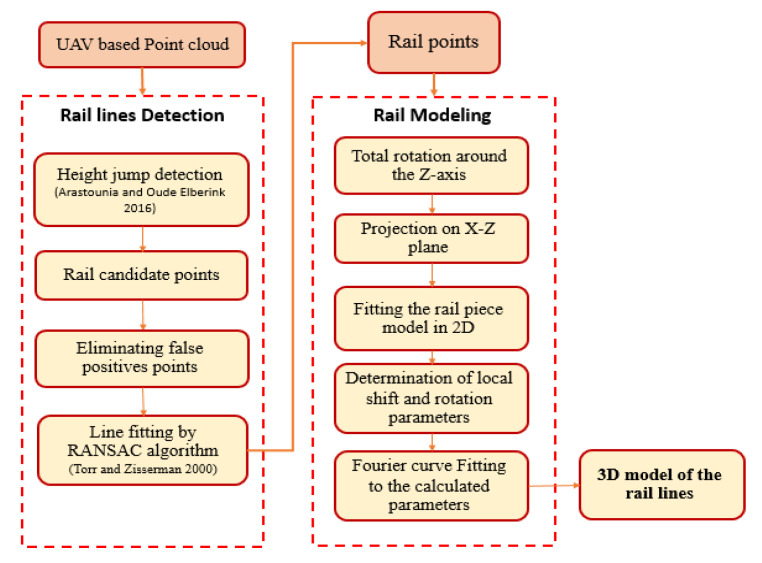
Flowchart of the proposed method.

Our goal in this study is to detect and model railway lines using a colored point cloud obtained from the photogrammetric method. With the ability to capture images with high overlap and at low altitudes, UAVs make it possible to produce dense and high-resolution point clouds using the structure from motion (SfM) algorithm [25]. This algorithm identifies and matches the key points in the overlapping area in each pair of images taken from the study area. Then, using the iterative bundle adjustment algorithm, the relative orientation parameters of the cameras are calculated, and a sparse point cloud is created. Finally, the multi-view stereo (MVS) technique is employed to construct a dense point cloud [26]. This point cloud is used to model rail lines according to the proposed method illustrated in Figure 1.

The proposed method consists of the following two major steps:

(1) Identifying and extracting the points related to the rail lines.
(2) 3D modeling of the extracted points.

In the following sections we will discuss the details of each step.

### 3.1. Rail Line Detection

As mentioned in Figure 1, the first step of the detection of the rail point from all points in the point cloud is height jump detection. For this purpose, we used the method presented by Arastounia et al. [13]. In this method, a 2D planimetric grid is first fitted to the points. Next, the cells whose height variations exceed the height of the rail tracks are selected. These height variations of each grid cell are obtained by calculating the height difference between the 95th and 5th percentiles of the heights of the points in which, accordingly, the noise and outliers are filtered out. Finally, the points higher than the 90th percentile of the remaining points in the selected cells are marked as rail candidate points (assuming that the track bed points make up a large part of each neighborhood’s points (90%)). However, due to the presence of other objects, such as walls and cars, and due to height changes resulting from the ground slope in the rail bed, multiple wrong points are selected as rail candidates (i.e., false positives). Therefore, in addition to considering the height of the points, other conditions are also included to improve the results. Due to the sunlight direction during the imaging and considering the shadows formed along rail tracks, a minimum intensity of points is considered in each grid cell. Due to the low intensity in shaded areas, the cells in which the minimum intensity (according to Equation (1)) is higher than the specified threshold are removed from the candidate points. The second condition is about one of the geometric features of the railway line, i.e., planarity, whose value is calculated according to Equation (2) [27].

(1) $$I=\frac{R+G+B}{3}$$

(2) $$Planarity=\frac{\lambda_{2}-\lambda_{3}}{\lambda_{1}}$$

where $\lambda_{i}$ are the eigenvalues of covariance matrix in descending order ($\lambda_{1}$ > $\lambda_{2}$ > $\lambda_{3}$), which is calculated with principal component analysis (PCA). In the mathematical definition, PCA is an orthogonal linear transformation that takes data to a new coordinate system so that the largest variance of the data is on the first coordinate axis, the second largest variance is on the second coordinate axis, and so on [28]. According to the dimensions of the data here and then the dimensions of the covariance matrix, three eigenvalues are calculated in each local neighborhood. If *A* is the covariance matrix of points in a local neighborhood, the eigenvalues (*λ*) of this matrix are obtained by solving Equation (4).

(3) $$A=\left(\begin{array}{ccc} \sigma_{x}^{2} & \sigma_{xy} & \sigma_{xz}\\ \sigma_{yx} & \sigma_{y}^{2} & \sigma_{yz}\\ \sigma_{zx} & \sigma_{zy} & \sigma_{z}^{2} \end{array}\right)$$

(4) |A−λI|=0

According to safety regulations, the railway bed is usually designed in such a way to have the lowest amount of local height variation in the longitudinal direction of the track. Therefore, the smallest eigenvalue $\lambda_{3}$ in a local neighborhood that does not have a piece of rail will be close to zero. Ideally this value should be zero, but due to the presence of ballasts in the rail bed and small changes in their height, a small value is chosen (here, 0.01). Additionally, in the area with a piece of rail, the smallest eigenvalue is higher than zero [14]. As a result, the value of the planarity feature in the grid cells containing the rails will be low and close to zero, and the points in which the value of this feature is higher than the threshold will be removed from the candidate rail points in the previous step. By applying these two conditions, the selected points’ likelihood of being rail candidates will be improved. In the next step, only the points that have a linear structure (which are the same as the rail lines) should remain. For this purpose, the M-estimator sample consensus (MSAC) algorithm, which is an extension of the RANSAC algorithm, is used [29]. This algorithm, which is a powerful estimation method for model fitting purposes, can find the line that best fits the valid points. Two parameters are used here: one is the maximum distance from the points to the line, and the other is the degree of the polynomial function. Since the rail track might be curved in medium-range distances, a straight line cannot be an optimized model as a best fit to the points. Therefore, a second-degree polynomial is fitted to the rail candidate points using the MSAC [29] algorithm. This curve, as a set of lines, eventually defines the rail tracks. Then, the points that are in a specified buffer from the fitted curve are considered as rail points.

### 3.2. 3D Modeling of Rail Lines

**Figure 2:**
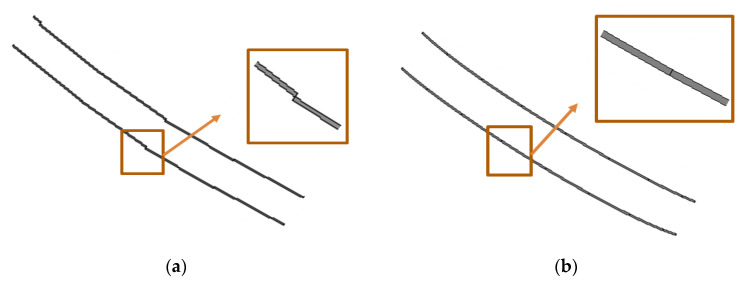
The 3D model of rail track 1 with (**a**) initial parameters and (**b**) interpolated parameters.

In this paper, a model-based method is used to reconstruct three-dimensional rail lines. In model-based reconstruction, a general pattern that includes the geometric features expected to be seen in the data is used as the initial model, and its parameters are calculated in a way that best fits the data [30]. Unlike data-based methods which require high point density in order to achieve the correct three-dimensional model, the model-based methods are suitable for lower density data or data that include noises and gaps (i.e., fewer inlier points). Additionally, the final models in this method have the correct topology [31]. The three-dimensional rail model includes a set of rail pieces that are defined by several positions and orientation parameters. The length of the rail model piece would be the same size as the fitted RANSAC lines in the previous step. The process of the model fitting is performed in several patches, which are equal to the length of the RANSAC line segments. To increase the accuracy of positioning, especially in places with limited point density, we utilize a “projection-based” method in order to fit the initial model. In this method, first, the three-dimensional points are projected to the XZ plane. The most important advantage of this process is the reduction of one dimension of the data and the process of determining the optimal position of the model in a two-dimensional space [32]. Due to the direction of the rail track and its angle with the *Y* axis, in order to project the points correctly and show the rail cross section in the front view, the overall rotation is applied around the Z and X axis as $\alpha_{z}$ and $\alpha_{x}$, respectively. The value of these rotations is obtained by calculating the angle of the fitted RANSAC line in each patch with the Y and X axes of the points. Here, to maintain the parallelism of the two rail lines, the average of the two fitted lines in each patch is considered. After the complete rotation of the points with the calculated angle and projecting them onto the XZ plane, the two-dimensional pre-defined model of the rail piece is fitted to the points. The model’s position is determined based on the maximum number of points in a specific buffer of the model. Since the rail points are scattered as wide as the width of the rail line, the RANSAC lines do not fit precisely in the centerline of the railhead. As a result, to determine the exact position and improve the RANSAC lines, the partial $\Delta \alpha_{z}$ and $\Delta \alpha_{x}$ rotations are also applied around the Z and X axes. These rotations are performed in a small interval of about 3 degrees with step sizes of 0.3 degrees. The best value of the rotation angle is also selected based on the rule of maximum points around the fitted model. After specifying the corrected rotation and shift parameters of the model, by adding the Y value of the desired patch, the model is transformed into the three-dimensional space. This process is conducted for all of the patches. However, fitting the rail piece model by the projection-based method cannot guarantee the continuity and smoothness of the final model of the rail lines, as shown in Figure 2a. For this reason, in the next step, each of the calculated parameters is interpolated using Fourier curve fitting. The Fourier series is defined as Equation (5).

(5) $$P_{j}\left(i\right)=\overset{n}{\underset{k=0}{\sum }}\left(a_{k}coskwi+b_{k}sinkwi\right)$$

where $P_{j}$: *j* = 1…5 is the calculated parameters of the model including X, Y, Z, $\Delta \alpha_{z}$ and $\Delta \alpha_{x}$. i is the number of patches, $a_{k}$, $b_{k}$, and w are the unknown coefficients of the interpolation function, and *n* is the degree of the Fourier curve. By calculating $P_{j}$ for all of the patches, a system of equations will be formed, which will then be used to solve the unknown coefficients. The estimated coefficients minimize the difference in the sum of squares of parameters in each patch and the interpolated parameters [11]. Using the new parameters found by this function, a uniform model of the entire rail track is obtained (Figure 2b).

**Figure 3:**
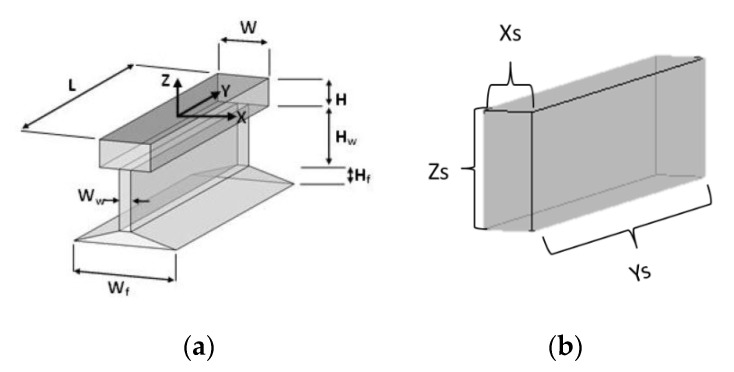
Rail piece model; (**a**) the original model [11] and (**b**) the model considered in this research.

The model of a rail segment can be defined by at least seven shape parameters, as shown in Figure 3a. Due to their nature in data collection, laser scanners provide more details of rail lines. On the other hand, the data obtained from aerial UAV images only show the general outline of railway points. Additionally, due to the quality of the obtained point cloud, all details of the model, i.e., seven parameters, cannot be recovered and therefore, here, a simpler model containing three parameters (cf. Figure 3b) are considered to be extracted. The local coordinates of this model are defined by three transfer parameters and two rotational parameters. The use of project-based methods for fitting the model reduces one of the rotational parameters and facilitates the process.

## 4. Results and Discussion

### 4.1. Data Acquisition

**Figure 4:**
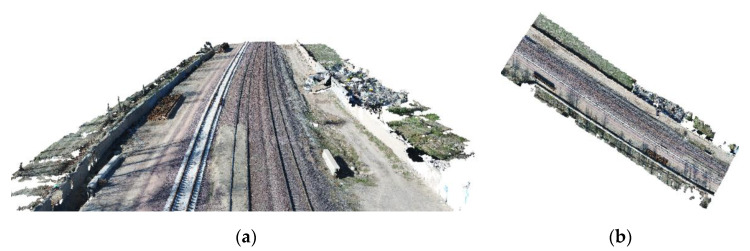
UAV-based point cloud from (**a**) the perspective view and (**b**) top view.

The data which are used here to evaluate the proposed method include UAV images from the Garmdareh region of the Alborz province in Iran and comprise about 200 m of railway lines. The images are taken using DJI Phantom 4 Pro with a sensor that can capture an image size of 5472 × 3648 pixels. The altitude of the flight is about 30 m. The ground sampling distance (GSD) of the images and also the point spacing of the generated point cloud are about 8mm. Due to the need for high ground resolution to identify rail lines, low flight altitudes are considered. The key points are first identified and matched using the scale-invariant feature transform (SIFT) operator in the overlapping parts of the images, and the relative orientation of the images is performed. Then, using the method of dense image matching with ground control points, the absolute orientation of the images is performed, and the dense point cloud of the region is generated, as shown in Figure 4. These steps are performed using the Agisoft PhotoScan software package [33].

The desired inspected rail area includes a length of about 1 km of rail tracks. To assess the accuracy of the generated point cloud, we use eight control points and two checkpoints in which their coordinates are measured by GPS. The root mean square errors (RMSEs) of the residuals at the control points and checkpoints are listed in Table 1.

A part of the data (about 100 m) is used to identify and model the railway lines.

### 4.2. Rail Line Detection and Modeling

**Figure 5:**
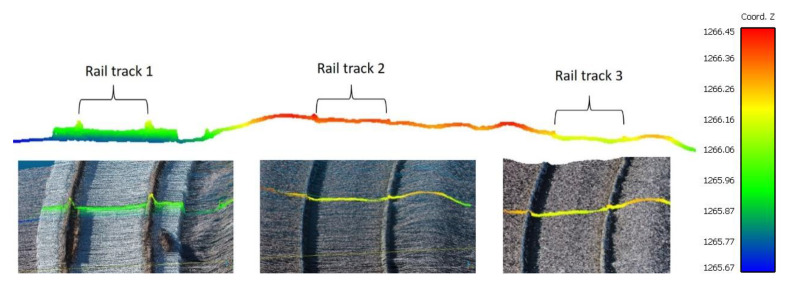
Cross section of railroad tracks that are colored according to the relative height of the points.

The first step is to identify the rail lines at the point cloud by height jump detection. There are three separate rail tracks existing in the study area next to each other. As we can see in Figure 5, which shows the cross section of the rail tracks, the area around railways 2 and 3 is almost fully covered by the ballast and, therefore, the rail lines are not “obviously” higher than the neighboring ground points. Accordingly, the number of detected rail points on these two tracks is minimal. So, detection and modeling, especially in these two railway tracks, is one of the challenges which we are facing in this research.

**Figure 6:**
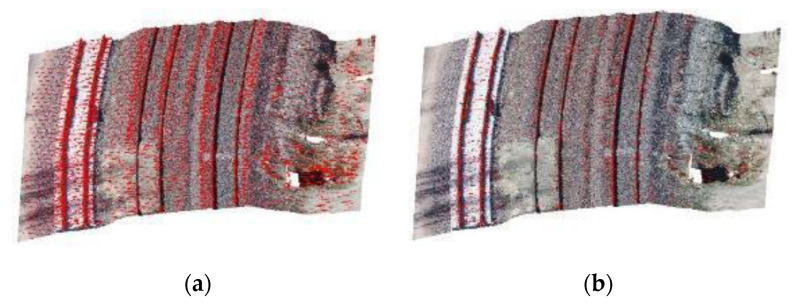
Extraction of rail candidate points (red) by height jump detection; (**a**) before and (**b**) after intensity and planarity conditioning.

Accordingly, it is necessary to consider a small amount for the threshold of the height variation of each cell so that rail lines 2 and 3 can be identified. Based on data analysis, a 4 cm threshold is considered as a good value for the height difference between the points and the rail bed. Another important parameter at this stage is the size of the neighboring cells. This value must be set so that it does not contain two rail lines at the same time. It should also not be so small that it does not include the features needed to identify candidate rail points [34]. Given the standard gauge of railway lines [35] (1.435 m), here, we consider a value of 1 m for the number of cells. To calculate the “planarity” feature, the spherical neighborhood radius is selected in the same way. After the height jump detection, the false positive points are removed by adding the two conditions of intensity and planarity. Figure 6 shows the rail candidate points before applying these two conditions, in which the non-rail points are selected incorrectly due to the slope of the ground and a bulge in the rail bed. These points, which are incorrectly selected as rails, due to geometric similarities to rail tracks (being linear), cause trouble for line fitting in the next step. Figure 6b shows the results of the stage of identifying rail candidate points after applying the above two conditions.

**Figure 7:**
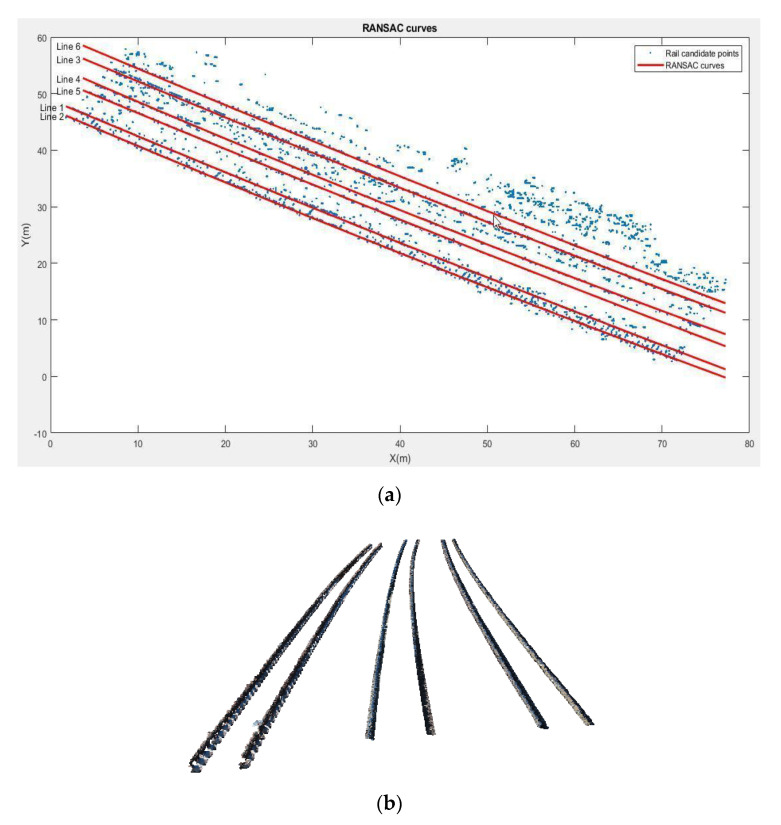
Result of curve fitting; (**a**) RANSAC polynomial and (**b**) rail tracks points.

In the next step, the RANSAC curve is fitted to the rail candidate points (Figure 7a) to extract the objects with a linear structure, like the rails. Since the rail track is curved, and for the lines to have the best fit to the centerline of the railhead, we consider a second-degree polynomial using the RANSAC algorithm to fit the points. This curve contains the straight lines created by connecting all the inliers. However, due to the low curvature of the railways, this number of points is not required to form a curve. So, we use the Douglas–Peuker method [36] to simplify the curve. Figure 7 shows the points extracted as rails after the curve fitting stage. This algorithm is an iterative method that converts a curve composed of linear parts into a similar curve with fewer points. In this algorithm, the degree of dissimilarity is defined based on the maximum distance between the main curve and the simplified curve. By applying this algorithm, the RANSAC curve is simplified and a limited number of line segments remain. By calculating the orientation of each line segment, the total rotation angles are obtained. Due to the fact that the remaining points in the simplified curves are more likely to be in the fractured and curved areas, the length of the RANSAC lines is longer in the straight sections of the track and less in the curved areas. Thus, the length of the patches and the model pieces also depends on the changes in the curvature of the track. Figure 7 shows the points extracted as rails after the curve fitting stage.

**Figure 8:**
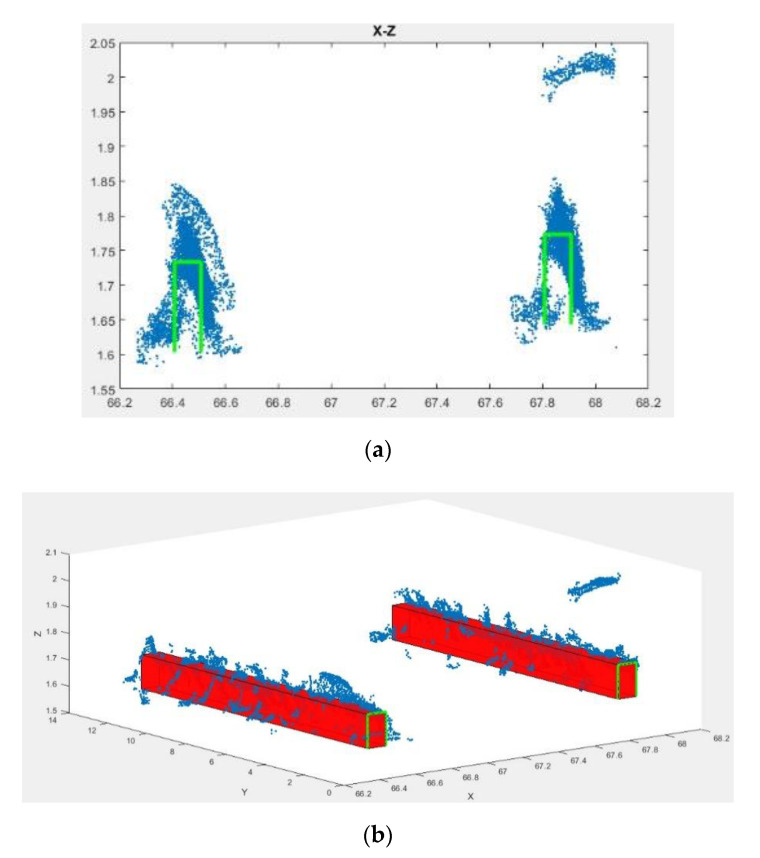
The positioning of the rail piece model for track 1 (**a**) in 2D and (**b**) 3D space.

These points are first divided into patches based on the length of the RANSAC lines. Then, each patch is sequentially rotated in two directions of x and z, represented by $\Delta \alpha_{z}\;$and $\Delta \alpha_{x},\;$as much as $\alpha_{z}+\Delta \alpha_{z}$ and $\alpha_{x}+\Delta \alpha_{x}$ to have an appropriate position relative to the XZ plane. By projecting the points on this plane, the position of the model is determined (Figure 8a) using the method described in Section 3.2.

**Figure 9:**
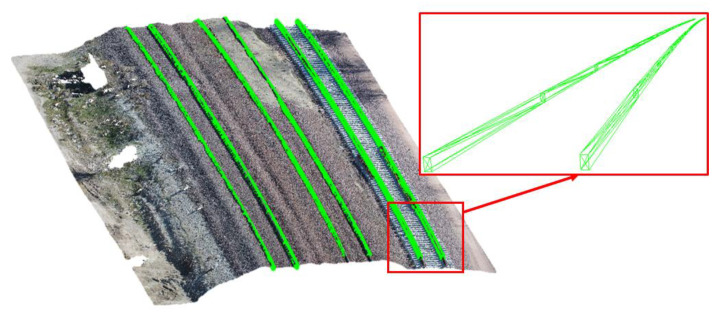
Final 3D model of rail tracks overlaid on the point cloud.

According to the design parameters, the values of the X and Z of the model for rail track 1 are 7 cm and 13 cm, respectively. For tracks 2 and 3, due to the presence of the surrounding ballast, the visible height of the model is considered to be 7 cm. After determining the two-dimensional position of the model, by adding the value of Y at the end of each piece, the models are transferred to the three-dimensional space (Figure 8b). In the final step, in order to integrate all pieces into each other to form a unified final model, each of the five model parameters is interpolated separately using the Fourier series. Therefore, in order to prevent oscillation and waviness in the final model, the use of higher degrees of the Fourier function is avoided [11]. The final three-dimensional model of rail tracks using the proposed method is presented in Figure 9.

### 4.3. Evaluation of Results

**Figure 10:**
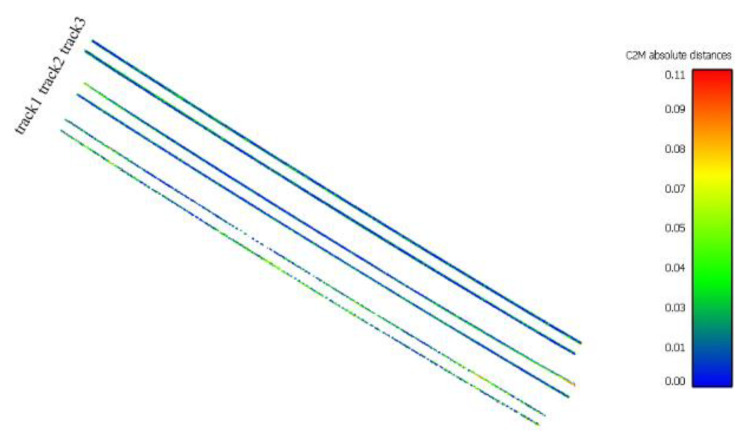
Distance between the point cloud and fitted models to the tracks.

In order to evaluate the results, the geometric distance of rail points from the created models is calculated, which is shown in Figure 10 with different colors. Some statistical parameters, such as mean, median, and standard deviation regarding the distance values, were obtained for a quantitative assessment (Table 2).

Generally, the mean value indicates the accuracy of the fitted model, but the outlier points in which their distances from the model are more than 10 cm affects the average value. Similarly, the median value is calculated for each track point. This value generally indicates less than 3 cm in the three tracks, which is the general accuracy of our modeling. The standard deviation also displays data focus around the average value. The lower the value, the higher the accuracy of the modeling process, and the better the final fit on the railhead points. Railways 2 and 3, as mentioned earlier, have a lower number of points and a more unfavorable distribution compared to railway 1, which is why their modeling accuracy is also slightly less than track 1. Track 3 has more outlier points, and due to the higher accumulation of ballast around it and the greater depression of these rail lines on the ground, fewer points of rail are identified. As a result, modeling accuracy in this track is less than for track number 2.

To evaluate the performance of the proposed method, a comparison is made with the method presented in [11] (Table 2). In this method, rail track modeling is performed using an MLS point cloud. The defined model contains seven shape parameters and six orientation parameters (Figure 3a). Determining the position of the model in three-dimensional space is done with the MCMC method. Although the point cloud obtained from the mobile laser scanner creates more detailed information of the rail track than the stereo matching method, the accuracy of our proposed projection-based method is higher in the case of modeling. The percentage of outliers in the tracks, which shows the accuracy of identifying rail points, is lower in our method. Only in track 3, due to improper scattering of points and more outlier points, is the median value of the geometric distance higher than the comparative method.

## 5. Conclusions and Discussion

Our aim in this study is to utilize the point cloud obtained from UAV photogrammetry to identify and model railway lines, as one of the essential urban infrastructures, so that we can take advantage of UAVs in this regard. Due to the nature of the data acquisition from the above, the UAVs do not produce higher quality data compared to terrestrial laser scanners in capturing the details and the side view of rails. Additionally, in rail environments, low altitude flights are required to detect tracks with high ground resolution. This could increase the complexity of the processing. However, UAVs have other advantages that make them efficient to use, including, for example, the ability to remotely capture data, reduce the risk of people being exposed to railroads and high speeds to inspect the raillines, and a relatively low cost of data retrieval.

In this study, we identified and modeled railway tracks using point clouds obtained from stereo matching methods in overlapping images from UAVs. For this purpose, we proposed a projection-based process to make model positioning easier and faster by removing one dimension of the data. The mapping of rail points on a two-dimensional plane increases the number of valid points to fit the model, and this can be useful in cases where the density of the desired object points is limited. Finally, our proposed method was able to achieve an acceptable result with a median geometric distance of the point to the model of less than 1.5 cm in two rail tracks. In tracks 1 and 2, the above result was able to improve on the results in [11]. In track 3, this accuracy was decreased due to the presence of more outlier points, but this value was still less than 3 cm. Although the second and third rail tracks were entirely covered by ballast and the railway points were difficult to identify, the proposed method in this article was able to model the railway lines successfully. Although a laser scanner’s points are inherently more accurate with more detail of rail tracks than image-based point clouds, our proposed projection-based method achieved a model accuracy close to or even better than the model produced with the MLS point cloud in [11]. This shows the applicability and high performance of the proposed method in modeling rail tracks despite the low quality and inadequate dispersion or low density of initial points.

The level of accuracy that was obtained in the proposed method is useful for rail applications such as asset inventory, visualization, and the detection of extensive track fractures. However, applications that need millimeter-level accuracy, e.g., detecting minor changes in paths, rail wear, etc., require higher quality data and more control in the data capturing and processing conditions, which of course increase the project costs and required time. In most of the model-driven methods used to model rail lines, complex and time-consuming calculations have been used, but here, the use of the projection-based approach in the process of fitting the model to the points was able to reduce the complexity of computations. Additionally, in the proposed method for the positioning of the model, there was no need to use approaches such as least squares, which is a non-linear method and requires time-consuming calculations. In the least squares algorithm, the sparsity of points affects the computation of the model orientation parameters and may cause the algorithm to fail. However, we used a method here that only determines the position of the model in two dimensions by counting the number of points around the model and selecting the location with the highest number of points. This method does not depend on the sparsity of points or their low density; in addition, it is simple and fast.

Finally, we briefly summarize the novelty of the proposed projection-based algorithm for 3D modeling of railways as the following key issues:

- By employing a projection-based algorithm and projecting the 3D point cloud onto the plane perpendicular to the main orientation of the rail segment, in fact, we eliminate one dimension and therefore we further process the modeling in 2D space instead of 3D space.
- Reducing one dimension also increases the speed of processing.
- Since one of the most challenging issues in UAV point clouds of rail tracks is the huge amount of noise, by projecting the point cloud into 2D space, we increase the amount of inlier points which alleviate the impact of the incompleteness of the data on the model fitting result, which creates a more robust 2D and then 3D model of the rail segment.
- Another innovative strategy in our rail track reconstruction approach is that, in considering the local linearity of the railroad structure, the extrusion of a 2D model to a 3D model is performed in such a way that the whole part of the railroad under investigation is split into some locally linear and smoothly connected patches.

As future research, this method needs to be applied to more complicated tracks, such as railways with turnouts, or even other structures such as guardrails, to examine the effectiveness of the proposed method further.

## Figures and Tables

**Table 1:** RMSE at control points and checkpoints.

Total Errors	X Error (cm)	Y Error (cm)	Z Error (cm)	Total (cm)	Image (pix)
Control points	4.6	6.3	4.7	9.2	0.7
Checkpoints	3.4	6.6	1.2	7.6	0.4

**Table 2:** Statistical parameters of point-to-model distance in each track and comparison with the method presented in [11].

	Rail Track	Mean (cm)	Standard Deviation (cm)	Median (cm)	Outliers (>10 cm) (%)
Proposed method	t1	1.68	2.37	1.1	0.01%
	t2	2.04	2.9	1.3	0.7%
	t3	3.5	4.68	2.7	3.2%
Method in [11]	A1/t1	10.08	N/A	1.67	4.4%
	A1/t2	3.53	N/A	1.43	3.8%
	A2/t1	6.13	N/A	1.93	5.1%
	A2/t2	3.64	N/A	1.99	3.7%

## References

[1] Jwa Y., Sonh G. (2015). Kalman Filter Based Railway Tracking from Mobile Lidar Data. ISPRS Ann. Photogramm. Remote. Sens. Spat. Inf. Sci..

[2] Liu S., Wang Q., Luo Y. (2019). A review of applications of visual inspection technology based on image processing in the railway industry. Transp. Saf. Environ..

[3] Zheng S., Chai X., An X., Li L. Railway track gauge inspection method based on computer vision. Proceedings of the 2012 IEEE International Conference on Mechatronics and Automation.

[4] Chekure E.T., Naudé K.A., Freere P. The effective use of the exhaustive search block matching algorithm in railway line tracking. Proceedings of the 2017 IEEE AFRICON.

[5] Wu H., Siu W.-C. Real time railway extraction by angle alignment measure. Proceedings of the 2015 IEEE International Conference on Image Processing (ICIP).

[6] Qi Z., Tian Y., Shi Y. (2012). Efficient railway tracks detection and turnouts recognition method using HOG features. Neural Comput. Appl..

[7] Wang Z., Cai B., Chunxiao J., Tao C., Zhang Z., Wang Y., Li S., Huang F., Fu S., Zhang F. Geometry constraints-based visual rail track extraction. Proceedings of the 2016 12th World Congress on Intelligent Control and Automation (WCICA).

[8] Uribe J.A., Fonseca L., Vargas J. Video based system for railroad collision warning. Proceedings of the 2012 IEEE International Carnahan Conference on Security Technology (ICCST).

[9] Beger R., Gedrange C., Hecht R., Neubert M. (2011). Data fusion of extremely high resolution aerial imagery and LiDAR data for automated railroad centre line reconstruction. ISPRS J. Photogramm. Remote. Sens..

[10] Fischler M., Bolles R. (1981). Random sample consensus: A paradigm for model fitting with applications to image analysis and automated cartography. Commun. ACM.

[11] Elberink S.O., Khoshelham K., Arastounia M., Benito D.D. (2013). Rail Track Detection and Modelling in Mobile Laser Scanner Data. ISPRS Ann. Photogramm. Remote. Sens. Spat. Inf. Sci..

[12] Zhu L., Hyyppä J. (2014). The Use of Airborne and Mobile Laser Scanning for Modeling Railway Environments in 3D. Remote. Sens..

[13] Arastounia M., Elberink S.O. (2016). Application of Template Matching for Improving Classification of Urban Railroad Point Clouds. Sensors.

[14] Arastounia M. (2017). An Enhanced Algorithm for Concurrent Recognition of Rail Tracks and Power Cables from Terrestrial and Airborne LiDAR Point Clouds. Infrastructures.

[15] Ahmed M., Guillemet A., Shahi A., Haas C.T., West J.S., Haas R.C. Comparison of point-cloud acquisition from laser-scanning and photogrammetry based on field experimentation. Proceedings of the CSCE 3rd International/9th Construction Specialty Conference.

[16] Alidoost F., Arefi H. (2017). Comparison of Uas-Based Photogrammetry Software for 3d Point Cloud Generation: A Survey over A Historical Site. ISPRS Ann. Photogramm. Remote. Sens. Spat. Inf. Sci..

[17] Malihi S., Zoej M.J.V., Hahn M., Mokhtarzade M. (2018). Window Detection from UAS-Derived Photogrammetric Point Cloud Employing Density-Based Filtering and Perceptual Organization. Remote. Sens..

[18] Pan Y., Dong Y., Wang D., Chen A., Ye Z. (2019). Three-Dimensional Reconstruction of Structural Surface Model of Heritage Bridges Using UAV-Based Photogrammetric Point Clouds. Remote. Sens..

[19] Kerle N., Nex F., Gerke M., Duarte D., Vetrivel A. (2019). UAV-Based Structural Damage Mapping: A Review. ISPRS Int. J. Geo-Inf..

[20] CYBERHAWK. https://thecyberhawk.com/cyberhawk-discuss-the-advantages-of-uavs-to-the-rail-industry/.

[21] Falamarzi A., Moridpour S., Nazem M. (2019). A review on existing sensors and devices for inspecting railway infrastructure. J. Kejuru..

[22] Páli E., Mathe K., Tamas L., Buşoniu L. Railway track following with the AR. Drone using vanishing point detection. Proceedings of the 2014 IEEE International Conference on Automation, Quality and Testing, Robotics.

[23] Singh A.K., Swarup A., Agarwal A., Singh D. (2019). Vision based rail track extraction and monitoring through drone imagery. ICT Express.

[24] Banić M., Miltenović A., Pavlović M., Ćirić I. (2019). Intelligent Machine Vision Based Railway Infrastructure Inspection and Monitoring Using Uav. Facta Univ. Series Mech. Eng..

[25] Westoby M., Brasington J., Glasser N.F., Hambrey M., Reynolds J. (2012). ‘Structure-from-Motion’ photogrammetry: A low-cost, effective tool for geoscience applications. Geomorphology.

[26] García-Luna R., Senent S., Jurado-Piña R., Jimenez R. (2019). Structure from Motion photogrammetry to characterize underground rock masses: Experiences from two real tunnels. Tunn. Undergr. Space Technol..

[27] Hackel T., Wegner J.D., Schindler K. Contour detection in unstructured 3d point clouds. Proceedings of the IEEE Conference on Computer Vision and Pattern Recognition.

[28] Jolliffe I.T. (2002). Principal Component Analysis.

[29] Torr P., Zisserman A. (2000). MLESAC: A New Robust Estimator with Application to Estimating Image Geometry. Comput. Vis. Image Underst..

[30] Schindler K., Bauer J. A model-based method for building reconstruction. Proceedings of the First IEEE International Workshop on Higher-Level Knowledge in 3D Modeling and Motion Analysis (HLK 2003).

[31] Sohn G., Dowman I. (2007). Data fusion of high-resolution satellite imagery and LiDAR data for automatic building extraction. ISPRS J. Photogramm. Remote. Sens..

[32] Arefi H., Reinartz P. (2013). Building Reconstruction Using DSM and Orthorectified Images. Remote. Sens..

[33] AgiSoft PhotoScan Professional (Version 1.4). https://www.agisoft.com.

[34] Arastounia M. (2015). Automated Recognition of Railroad Infrastructure in Rural Areas from LIDAR Data. Remote. Sens..

[35] Iran Ministry of Roads and Urban Development (2005). Railway Track Super Structure General Technical Specifications.

[36] Douglas D.H., Peucker T.K. (1973). Algorithms for the Reduction of the Number of Points Required to Represent a Digitized Line or Its Caricature. Cartogr. Int. J. Geogr. Inf. Geovisualization.

